# High Amylose White Rice Reduces Post-Prandial Glycemic Response but Not Appetite in Humans

**DOI:** 10.3390/nu7075225

**Published:** 2015-07-02

**Authors:** Alison M. Zenel, Maria L. Stewart

**Affiliations:** Department of Human Nutrition, Food and Animal Sciences, University of Hawaii at Manoa, Honolulu, HI 96822, USA; E-Mail: mstew@hawaii.edu

**Keywords:** high amylose, short grain rice, dietary fiber, appetite, visual analog scale, glucose, insulin

## Abstract

The present study compared the effects of three rice cultivars on postprandial glycemic control and appetite. A single-blind, randomized, crossover clinical trial was performed with 18 healthy subjects, nine males and nine females. Three treatments were administered at three separate study visits: commercially available conventional white rice (short grain), specialty high amylose white rice 1 (Dixiebelle), and specialty high amylose white rice 2 (Rondo). Postprandial capillary blood glucose, venous blood glucose and insulin measurements, and appetite visual analog scale (VAS) surveys were done over the course of two hours. The capillary blood glucose concentrations were significantly lower for Rondo compared to short grain rice at 30 min, and for Dixiebelle and Rondo compared to short grain rice at 45, 60, and 120 min. Capillary blood glucose area under the curve (AUC) was significantly lower for Dixiebelle and Rondo compared to short grain rice. Subjects were significantly more hungry at 30 min after Dixiebelle intake than Rondo intake, but there were no other significant effects in appetite ratings. The present study determined that intake of high amylose rice with resistant starch (RS) can attenuate postprandial blood glucose and insulin response in comparison to short grain rice.

## 1. Introduction

Rice consumption in the United States has increased in recent decades from 27% of adults reporting consumption of >1/4 oz of rice per day (NHANES 1999–2004) [1] to 84% of adults (NHANES 2005–2010) [2]; however, daily intake is still relatively low, with 59% of adults consuming 0.25 to 0.5 oz equivalents of rice per day (NHANES 2005–2010). Globally, rice intake varies widely per capita, from 5.2 kg/year in Europe to 77.2 kg/year in Asia [3]. With trends of increasing rice consumption, it is necessary to understand the differences in carbohydrate availability among the different cultivars of rice.

Rice is typically considered a highly digestible source of carbohydrate, but rate of digestion and the resulting glycemic response varies among rice cultivars and preparation techniques [4,5]. Amylose content of rice affects glycemic response due to the tendency of amylose to retain its crystalline structure after cooking. This reduces enzyme accessibility, and results in greater proportions of slowly-digestible starch and resistant starch (RS). A study of sixteen rice cultivars grown in the United States reported wide ranges in amylose content (0.3%–31.3% dry weight basis (dwb)), slowly digestible starch (13.6%–23.3% dwb) and resistant starch (2.0%–6.7% dwb), despite the total starch content being highly uniform (82.2%–86.6% dwb) among the cultivars [6]. This study previously reported that the rapidly digestible, slowly digestible starch and resistant starch content of Dixiebelle and Rondo to be 61.7%, 23.2%, 4.7% and 60.9%, 16.8%, 6.3% dwb, respectively. When compared to low-amylose rice, the starch profiles were not consistently, significantly different. The variations in starch characteristics translated to differences in *in vitro* digestibility among the cultivars.

Resistant starch, a type of dietary fiber, is defined as all types of starch and starch degradation products that resist digestion and absorption in the small intestine when consumed, and enter the large intestine [7,8]. Resistant starch is associated with many health benefits such as improved glycemic control, improved insulin sensitivity, improved digestive health and weight management (as reviewed by [9]). Rice with high RS content has the potential to exhibit the beneficial physiological effects associated with RS intake. This is of particular interest, as rates of obesity, diabetes, and cardiovascular disease are increasing worldwide. Further research is necessary to understand the variations in health outcome within a single type of food, for example, rice.

The objective of this randomized, single-blind, crossover clinical trial study was to investigate the effects of white (milled) rice of two cultivars having high RS on postprandial blood glucose, insulin, and appetite in healthy adults, compared to a control short-grain conventional white rice. Palatability of the rice cultivars was also investigated. It was hypothesized that high RS rice consumption would result in lower blood glucose and insulin concentrations and would be more satiating, compared to the control rice.

## 2. Experimental Section

### 2.1. Subjects

**Figure 1:**
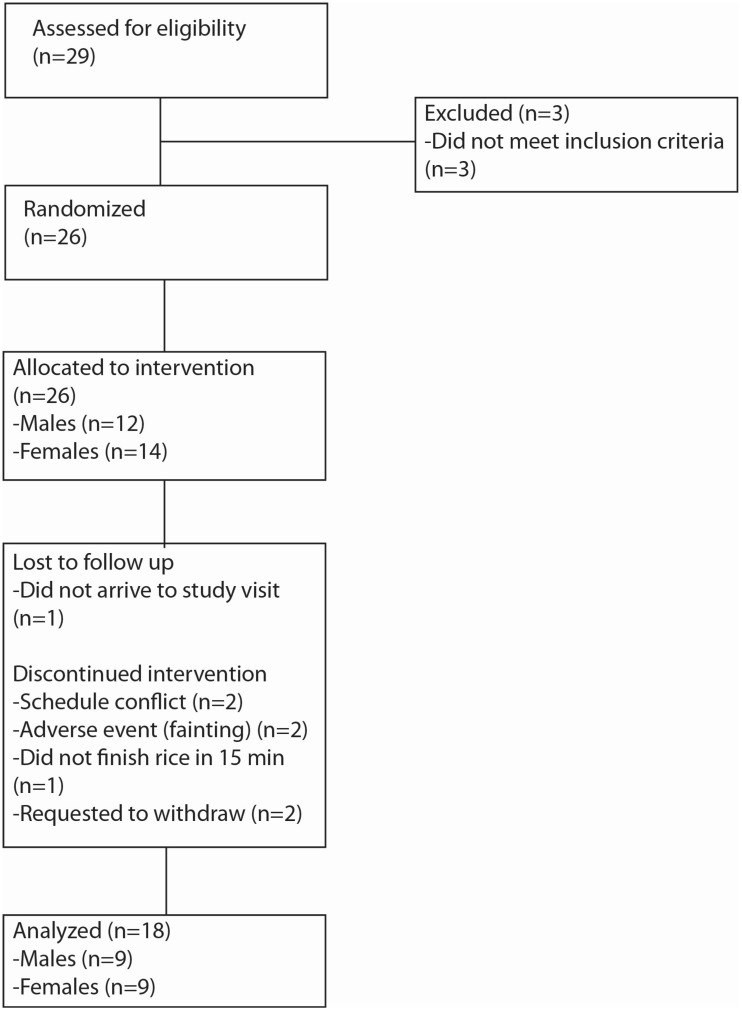
Flow diagram depicting the passage of subjects through the intervention.

All aspects of this study were approved by the University of Hawaii at Manoa Institutional Review Board (approval 5/6/2012, CHS #19457) and registered through clinicaltrials.gov (registration #NCT01685879). To recruit subjects, fliers were placed around the University of Hawaii at Manoa and Queen’s Medical Center. Subjects were screened by telephone or email for initial eligibility. After passing the initial screening, subjects attended a screening visit to complete a health history questionnaire to confirm eligibility and receive study materials. Inclusion criteria included general “good health”, 18–40 years old, non-vegetarian, non-smoking, BMI < 30 kg/m^2^, a habitual breakfast eater, habitually eats rice, able to fast for 12 h, and available in the morning on weekdays for study visits. Exclusion criteria included diagnosis of diabetes mellitus (type I or II), hyper- or hypoglycemia, hyperinsulinemia, history of gastrointestinal disease, and history of disordered eating. A subject was also excluded if she was pregnant or lactating, he or she was taking any medications that controlled blood glucose or insulin, appetite, or weight, or if the subject had any known food allergies. The necessary sample size to detect difference in serum insulin response is 18 subjects based on a standard power calculation (α = 0.05 and β = 0.80). Twenty six subjects were enrolled in the study and 18 subjects (9 males and 9 females) completed all of the study visits (Figure 1).

### 2.2. Treatments

The high amylose rice cultivars containing high RS, Dixiebelle and Rondo, were obtained from the USDA Dale Bumpers National Rice Research Center in Stuttgart, Arkansas. The amylose content of the selected rice cultivars was previously reported Dixiebelle (30.3% dsb) and Rondo (29.0% dsb) [6]. The conventional short grain white rice (control) used was Tamanishiki brand, available at grocery stores in the Honolulu, HI area. Rice samples were prepared using a conventional rice cooker (Aroma brand, San Diego, CA, USA). Rice (1/2 cup US) and water (3/4 cup US) were cooked until automatic completion in the rice cooker (approximately 15 min). Portion sizes were matched for volume (1¼ cups US, 300 mL). After rice preparation, the rice treatments were stored in a refrigerator until the study visit. Rice was prepared up to 3 days in advance and stored in a refrigerator at 4 °C prior to the study visits. The rice samples were reheated in a microwave for 1 minute on high power immediately before consumption. Subjects consumed the rice in a paper bowl with a plastic spoon, with no other ingredients. Throughout the study visit, subjects were allowed to drink water. Resistant starch content of the three rice samples was confirmed by AOAC official method 2002.02 using a commercially available assay kit (Megazyme International, Wicklow, Ireland). Rice samples (tripiclate) were cooked, cooled to 4 °C and stored overnight, and reheated prior to RS assay.

### 2.3. Study Visits

Subjects completed a total of three study visits, and treatment order was randomly assigned at the time of enrollment. Subjects were blinded to the treatment identities. Study visits were held on weekday mornings. The day prior to the study visit, subjects kept a 1-day food record to document habitual diet. The subjects were instructed verbally on how to keep a food record by study staff, and the food record also included instructions and an example food record. Subjects began fasting at midnight (except for water), and were instructed to drink plenty of water the night before and the morning of the visit to assist with venous blood draws. Subjects arrived fasted for a minimum of 8 h.

**Figure 2:**
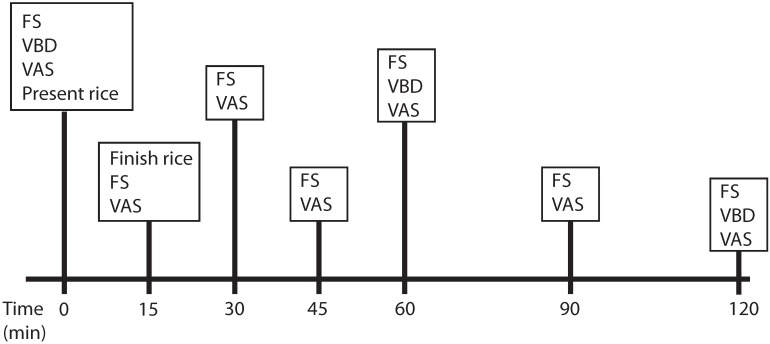
Timeline of the study visits. Abbreviations: FS, Finger stick blood draw for capillary glucose measurement; VBD, venous blood draw for glucose and insulin measurement; VAS, visual analog scale.

Each visit lasted approximately 2.5 h. A timeline of a typical study visit is shown in Figure 2. Height and weight measurements were taken upon arrival to the study visit. The study staff reviewed the food records with each subject to confirm all items consumed prior to the study visit. At time 0 min, the subjects had a venous blood draw, a capillary glucose measurement via finger stick, and completed a VAS to assess baseline appetite. Immediately following these measurements, time started elapsing (time = 0 min), and subjects were presented with the rice sample with 16 oz bottled water. Subjects were required to consume the entire rice sample within 15 min. Time zero was started at the onset of consumption due to the expected rapidly digestible and digestible starch. Subjects were required to finish their rice within 15 min to standardize the remaining timecourse as “postprandial” for all subjects. Capillary glucose measurement via finger stick and VAS were completed at times 15, 30, 45, 60, 90, and 120 min. At times 60 and 120 min, a venous blood draw was taken. Palatability of the rice samples was evaluated with visual analog scale (VAS) at 15 min.

During the study visit, subjects read, used an electronic mobile device, or watched television. If necessary, a heating pad was used in between venous blood draws to aid with the draws. Subjects received refreshments and compensation after each study visit.

### 2.4. Visual Analogue Scales

Appetite was evaluated using a 100 mm VAS. After reading a question, subjects marked where they felt their answer belonged on a 100 mm line, using it as a scale (0 mm to 100 mm). Questions were: How hungry do you feel? Response options: Not hungry at all (0 mm) to I have never been more hungry (100 mm); How satisfied do you feel? Response options: I am completely empty (0 mm) to I cannot eat another bite (100 mm); How full do you feel? Response options: Not full at all (0 mm) to Totally full (100 mm); How much do you think you can eat? Response options: Nothing at all (0 mm) to A lot (100 mm). Flint *et al.* determined appetite VAS scores to be reproducible and valid for appetite research studies [10].

At time 15 min, palatability of the treatment was assessed by five characteristics, from bad (0 mm) to good (100 mm). These characteristics were visual appeal, smell, taste, texture, and overall pleasantness of the treatment given.

### 2.5. Blood Analysis

Blood glucose concentrations at 0, 15, 30, 45, 60, 90, and 120 min were measured by finger stick with a One Touch Ultra glucose meter (Life Scan, Inc., Milpitas, CA, USA). The blood samples from the venous blood draws at 0, 60, and 120 min were analyzed by Diagnostic Lab Services for blood glucose and insulin concentration. Capillary blood glucose area under the curves (AUC) were calculated using the trapezoid rule [11].

### 2.6. Food Record Analysis

The Nutrition Data System for Research (NDSR) 2012 (Nutrition Coordinating Center, University of Minnesota, Minneapolis, MN, USA) was used to determine total nutrient and energy intakes for each food record. If a food was not listed in the NDSR program, the best match for the food was selected.

### 2.7. Statistical Analysis

Data were analyzed with SAS statistical software (Version 9.3, SAS Institute, Cary, NC, USA). Results are presented as mean ± standard error of the mean. Treatment effects on biochemical parameters, appetite ratings, and dietary intake were determined using “PROC MIXED” procedure for analysis of variance to control for subject variation. Dietary intake and visit order were also included as a predictors of biochemical parameters and appetite ratings to determine if prior intake affected the study visits. Treatment groups were compared using LSMeans. Significant differences were determined at *p* < 0.05.

## 3. Results

### 3.1. RS Content of Rice

The RS content of one portion (1¼ cup US) of rice was as follows: short grain white rice, 1.40 g RS; Dixiebelle rice 2.25 g RS; Rondo rice, 2.10 g RS.

### 3.2. Demographics

The demographic information on the study subjects is presented in Table 1. Subjects were young to middle aged adults (21–37 years). Female subjects’ mean BMI was in the “healthy” range while male subjects’ mean BMI was slightly above the “healthy” range.

**Table 1:** Study subject demographics.

Demographic	Total (*n* = 18)	Men (*n* = 9)	Women (*n* = 9)
**Age (years) (Mean, Range)**	26, 21–37	26, 25–30	27, 21–37
**Height (m) (Mean, Range)**	1.69, 1.57–1.88	1.74, 1.65–1.88	1.63, 1.57–1.73
**Weight (kg) (Mean, Range)**	66, 47–86	77, 68–86	57, 47–70
**BMI (kg/m^2^) (Mean, Range)**	23.2, 20.1–26.8	25.2, 23.9–26.8	21.2, 18.4–23.3

#### Dietary Intake

Dietary intake 24 h prior to study visits among treatments was not significantly different (Table 2).

**Table 2:** Previsit dietary intake.

	Short Grain Rice	Dixiebelle	Rondo	*p*-Value
**Total energy (MJ)**	7.75 ± 0.82	8.57 ± 0.51	9.24 ± 1.13	0.3251
**Total fat (g)**	74 ± 9	85 ± 8	94 ± 15	0.3581
**Total carbohydrate (g)**	215 ± 24	240 ± 16	241 ± 25	0.4629
**Total protein (g)**	81 ± 12	82 ± 8	97 ± 14	0.4579
**Total fiber (g)**	19 ± 2	20 ± 2	17 ± 2	0.5292
**Total available carbohydrate (g)**	196 ± 23	220 ± 15	222 ± 24	0.4339
**% energy from carbohydrate**	46 ± 2	47 ± 3	45 ± 2	0.7788
**% energy from fat**	34 ± 2	35 ± 2	36 ± 2	0.8196
**% energy from protein**	17 ± 1	16 ± 1	17 ± 1	0.7834
**% energy from alcohol**	2 ± 1	1 ± 1	2 ± 1	0.7590

Data are presented as mean ± SEM. *p*-Values calculated with Proc MIXED analysis of variance with LSMean comparison between treatments (*n* = 18).

### 3.3. Glucose Response

The mean capillary blood glucose concentration at 30 min was significantly higher for the short grain rice than Rondo rice (Table 3). At 45, 60, and 120 min, the mean capillary blood glucose concentrations were significantly higher for the short grain rice than both Dixiebelle and Rondo. In addition, the mean AUC for capillary blood glucose concentrations was significantly higher for the short grain rice than both Dixiebelle and Rondo. Finally, the mean venous blood glucose concentration at 60 min was significantly higher for the short grain rice than Rondo (Table 4). Previous total energy intake, total carbohydrate intake, total available carbohydrate, or treatment order intake did not influence glucose values when included in statistical model adjustment.

**Table 3:** Capillary blood glucose values (mg/dL, mean ± SEM) and AUC (mg*min/dL, mean ± SEM) before (0 min) and after treatment intake in healthy adults (*n* = 18).

	Short Grain Rice	Dixiebelle	Rondo	*p*-Value
**0 min**	94 ± 3	98 ± 2	94 ± 3	0.2791
**15 min**	99 ± 3	101 ± 4	98 ± 3	0.5769
**30 min**	137 ± 5 *	126 ± 5	122 ± 6	0.0555
**45 min**	144 ± 5 ^A^	127 ± 5 ^B^	129 ± 7 ^B^	0.0470
**60 min**	142 ± 7 ^A^ **	125 ± 6 ^B^ **	118 ± 5 ^B^ ***	0.0010
**90 min**	117 ± 4	112 ± 5	111 ± 3	0.4173
**120 min**	112 ± 4 ^A^ **	104 ± 4 ^B^ **	104 ± 2 ^B^ ***	0.0402
**AUC**	3519 ± 390 ^A^ **	2170 ± 371 ^B^ **	2419 ± 433 ^B^ ***	0.0063

* Within a row, cells with different superscript letters are significantly different (*p* < 0.05), Proc MIXED analysis of variance with LSMean comparison between treatments; ** Missing data for 1 subject; *** Missing data for 2 subjects.

**Table 4:** Venous blood glucose (mg/dL, mean ± SEM) and insulin (μIU/mL, mean ± SEM) values before (0 min) and after treatment intake in healthy adults (*n* = 18).

	Short Grain Rice	Dixiebelle	Rondo	*p*-Value
**Glucose, 0 min**	83 ± 2	83 ± 2	85 ± 1	0.3436
**Glucose, 60 min**	114 ± 6 ^A^ *	105 ± 6	103 ± 5 ^B^	0.0790
**Glucose, 120 min**	91 ± 3	95 ± 4	95 ± 2	0.3650
**Insulin, 0 min**	5.6 ± 0.7	6.1 ± 0.9	6.5 ± 0.7	0.4334
**Insulin, 60 min**	38.3 ± 6.7 ^A^ **	24.3 ± 3.8 ^B^	29.2 ± 6.3 ^B^ **	0.0009
**Insulin, 120 min**	21.0 ± 4.3 ^A^	15.2 ± 2.5 ^B^	18.4 ± 4.0	0.0467

* Within a row, cells with different superscript letters are significantly different (*p* < 0.05), Proc MIXED analysis of variance; ** Missing data for 1 subject.

### 3.4. Insulin Response

The mean venous insulin concentration at 60 min was significantly higher for the short grain rice than both Dixiebelle and Rondo (Table 4). At 120 min, the mean insulin concentration was significantly higher for the short grain rice than Dixiebelle. Previous total energy intake, total carbohydrate intake, total available carbohydrate, or treatment order intake did not influence insulin values when included in statistical model adjustment.

### 3.5. Appetite

**Figure 3:**
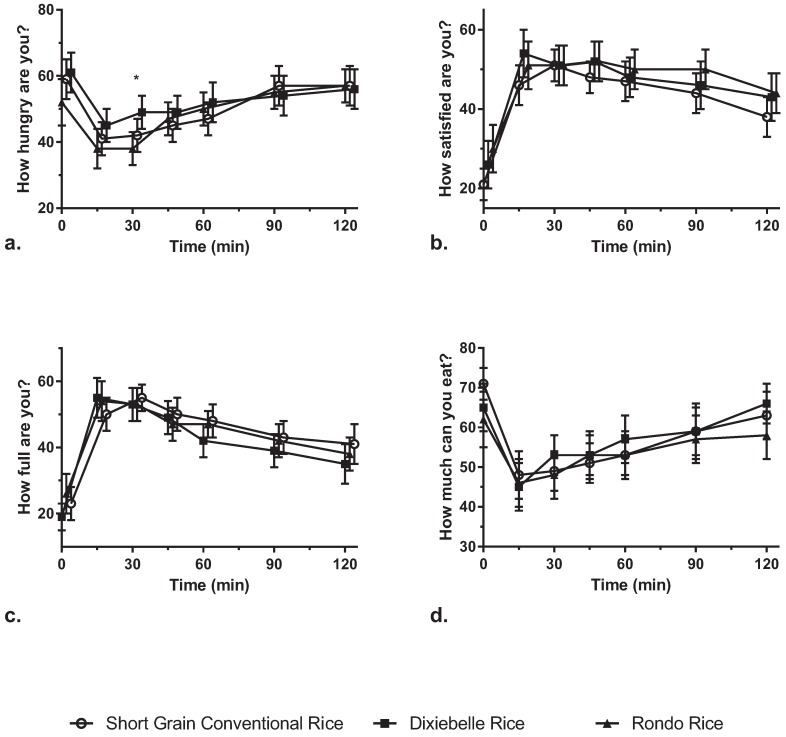
Appetite ratings. (**a**) Hunger “Not hungry at all” (0 mm) to “I have never been more hungry” (100 mm); (**b**) Satisfaction “I am completely empty” (0 mm) to “I cannot eat another bite” (100 mm); (**c**) Fullness “Not full at all” (0 mm) to “Totally full” (100 mm); (**d**) How much I can eat “Nothing at all” (0 mm) to “A lot” (100 mm). *p*-Values calculated with Proc MIXED analysis of variance with LSMean comparison between treatments.

Although treatments were not significantly different for most of the satiety measurements, only at 30 min, were subjects significantly more hungry after Dixiebelle intake than Rondo intake (Figure 3). Before treatments were administered at 0 min, subjects were significantly more satisfied before Rondo consumption than before short grain rice consumption. There were no significant differences among treatments when evaluating fullness or the ability to eat more. Previous total energy intake, total carbohydrate intake, total available carbohydrate, or treatment order intake did not influence appetite ratings when included in statistical model adjustment.

### 3.6. Palatability

The taste, texture, and pleasantness of the short grain rice were all significantly preferred over both Dixiebelle and Rondo (Table 5). There were no significant differences in visual appeal or smell among the rice treatments.

**Table 5:** Treatment palatability (mean ± SEM) from VAS assessment * in healthy adults (*n* = 18).

	Short Grain Rice	Dixiebelle	Rondo	*p*-Value
**Visual**	73 ± 3	68 ± 4	65 ± 5	0.1260
**Smell**	69 ± 3	64 ± 5	67 ± 5	0.5021
**Taste**	69 ± 3 ^A^ **	55 ± 5 ^B^	59 ± 5 ^B^	0.0421
**Texture**	75 ± 3 ^A^	52 ± 6 ^B^	56 ± 5 ^B^	0.0023
**Pleasantness**	73 ± 3 ^A^	65 ± 5 ^B^	63 ± 4 ^B^	0.0322

* Each characteristic was assessed as “bad” (0 mm) to “good” (100 mm); ** Within a row, cells with different superscript letters are significantly different (*p* < 0.05), Proc MIXED analysis of variance LSMean comparison between treatments.

## 4. Discussion

Solid foods containing RS have shown therapeutic effects on postprandial blood glucose response and insulin in healthy individuals. Slowly digestible starch content may also reduce postprandial glucose response. The high RS rice samples, when matched for volume, significantly attenuated postprandial blood glucose response with an RS dose amount of 2.25 g (Dixiebelle) and 2.10 g (Rondo), compared to the short grain rice (1.4 g). In a previous study, the RS dose per serving of rice that significantly improved postprandial blood glucose response in healthy adults (Chinese, nine males, seven females aged 23–26 years, BMI of 18–24 kg/m^2^) was 8.05 g RS, about 6 g more than the present study [12]. Therefore, an 8.05 g RS dose in a serving of rice may be unnecessary. However, Chiu *et al.* (2013) found that a 4.4 g RS dose in a serving of rice had no effects on postprandial blood glucose response in healthy adults (12 males, nine females, mean age 29, mean BMI of 22.9, ethnicity not stated) [13]. The effective RS dose in rice on postprandial blood glucose response needs to be further investigated, but it should be noted that RS dose may not be the only factor. High amylose rice also contains slowly digestible starch, which would contribute to blood glucose attenuation [6].

The present study also found that insulin response was significantly lower at 60 min with an RS dose of 2.25 g (Dixiebelle) and 2.10 g (Rondo), and at 120 min with the RS dose of 2.10 g (Dixiebelle), compared to the short grain rice. In the Li *et al.* (2010) study, the RS dose value of 8.05 g in a serving of rice significantly decreased insulin concentrations after intake starting at 45 min, then at 60, 90 and 120 min [12]. Because insulin concentration was not measured at 45 min in the present study, it is unknown whether Dixiebelle or Rondo affected insulin response at 45 min. In addition, the subjects in the present study were habitual rice eaters, determined at the initial screening for eligibility. A recent study investigated the effects of chronic intake of rice with added RS (derived from corn starch) on glycemic response in those with or at risk for type 2 diabetes [14]. The addition of 6.51 g RS added to short grain rice, daily, for four weeks significantly reduced postprandial glucose and insulin AUC, and fasting insulin. This study [14] suggests that the addition of RS has beneficial effects.

Prospective studies have noted a correlation between rice intake and increased risk of type 2 diabetes in multiple cohorts (as reviewed by [15]). Although this meta-analysis and systematic review reported a relative risk in the total population of 1.11 (1.08 to 1.14 95% CI, *p* for linear trend < 0.001), the range of intakes differed tremendously between Asian cohorts and Western cohorts. For example, the upper threshold for lowest quintile or quartile in the Chinese and Japanese cohorts reviewed by Hu *et al.* exceeded the lower threshold for the highest quintile or quartile in the Western cohorts [15]. Prospective studies included in Hu *et al.*’s review collected dietary intake using a food frequency questionnaire that did not differentiate the starch digestibility of the white rice samples used. As shown in the present study, white rice yields differing physiological effects, depending on the carbohydrate identity.

For other solid foods (muffins, bread, a nutrition bar, and corn cakes), a range from 6.5–15.6 g RS dose per serving significantly lowered postprandial blood glucose response and insulin response from 30 min to 2 h in healthy adults after intake [16,17,18,19,20]. However, Hallstrom *et al.* (2011) found a serving of bread (7.7 g RS) had no significant effects on insulin response in healthy adults (seven males, seven females, aged 20–35 years, mean BMI of 22.2 kg/m^2^, ethnicity not stated) [20]. Studies have also investigated the effects of intake of RS in solid foods on individuals with T2DM. Individuals with T2DM are afflicted with abnormally high fasting blood glucose concentrations, so postprandial blood glucose attenuation from RS intake may help with self-management. A treatment of rice with 7.8 g RS had no significant effects on postprandial blood glucose and insulin response in T2DM patients (seven males, five females, mean age 58, mean BMI of 30, ethnicity not stated, T2DM duration mean of four years) [21]. However, when subjects with untreated borderline T2DM (nine males, 11 females, mean age 50.5 years, fasting blood glucose 100–140 mg/dL, BMI not stated, Japanese) consumed bread with 6 g RS, postprandial blood glucose and insulin responses were significantly reduced [22]. Individuals with borderline T2DM may benefit from a lower dose of RS to see effects, while diabetic individuals may need a higher RS dose. Also, ethnicity may influence glycemic response. In the Yamada *et al.* (2005) study, subjects were Japanese, but ethnicity is not stated for the subjects in the Larsen *et al.* (1996) study [21,22]. However, in another study, subjects at risk for developing T2DM with insulin resistance (eight males, seven females, mean age 36, mean BMI of 37, African American) consumed bread with 12 g RS daily for 14 weeks, but no significant differences in blood glucose or insulin response were found [23]. The role of ethnicity in glycemic response maybe related to predisposition for diabetes, but further research is needed.

The RS dose in rice of the present study is much lower than the RS dose in the studies previously discussed. The inconsistency across food forms suggests that RS dose, alone, may not be relevant, and that food source and/or the proportions of digestible starch, slowly digestible starch, and RS may be more important.

Studies on RS in solid foods and its effects on appetite are limited. Willis *et al.* (2009) found that among muffins with different fiber types (RS, low fiber, corn bran, beta-glucan and oat fiber, polydextrose), the RS muffin (8 g RS) was the most satiating in healthy adults (seven males, 13 females, aged 18–65 years, mean BMI of 23, ethnicity not stated) [24]. However, in another study, there were no significant differences in appetite ratings after consumption of different fiber-type bars (RS (10 g), inulin, oligofructose, corn fiber) in healthy women (22 females, mean age 25, mean BMI of 23, ethnicity not stated) [25].

Few studies have investigated the effect of rice, without any added ingredients or meal items, on appetite. Chiu *et al.* (2013) found no significant differences in appetite between short grain rice and the rice containing 4.4 g RS in healthy adults [13]. In the present study, the only significant difference found was at 30 min, where subjects were more hungry after Dixiebelle treatment than after the Rondo treatment. Subjects preferred the short grain rice as compared to either of the long grain varieties Dixiebelle or Rondo for taste, texture, and pleasantness. All other ratings (fullness, satisfaction, how much can you eat) had no significant differences. However, subjects were significantly more satisfied before Rondo intake than before short grain rice intake, which may have influenced their appetite after treatment intake as well.

The high RS rice cultivars were chosen for this study based on potential for expanded commercial production. Patindol *et al.* (2010) compared the starch characteristics of 16 rice cultivars grown in the southern United States, at five different growing locations [6]. The RS amount in Dixiebelle and Rondo cultivars was stable across growing locations in Arkansas, Louisiana, Missouri, Mississippi, and Texas, while other cultivars were not stable in RS amounts. Additionally, a pilot sensory evaluation indicated that habitual rice consumers preferred Dixiebelle and Rondo cultivars over other high RS cultivars (unpublished data).

There are some limitations to this study. First, only three measurements of venous blood glucose and insulin concentrations were taken, thus AUC for venous blood glucose and insulin was not calculated. The study visit protocol was modified to have only three venous blood draws (at 0, 60, and 120 min) for subject comfort and study visit ease. Also, the study subjects were healthy individuals, with no issues in carbohydrate metabolism. The influence of slowly digestible starch content on the observed glycemic response is unknown, and while the present finding suggest a beneficial role in mediating blood glucose concentrations, further work is necessary to confirm this effect in individuals with pre-diabetes or diabetes.

## 5. Conclusions

The present study demonstrated that Dixiebelle rice and Rondo rice with higher levels of RS are capable of attenuating postprandial blood glucose and insulin response compared to short grain rice with lower RS in healthy individuals. The serving size of these rice samples was practical and can be easily incorporated into the diet. Future work should include an investigation on the RS dose in rice needed to have effects on postprandial glucose and insulin response in pre-diabetic and diabetic individuals.
